# Mitochondrial Regulation of NADPH Oxidase in Hindlimb Unweighting Rat Cerebral Arteries

**DOI:** 10.1371/journal.pone.0095916

**Published:** 2014-04-23

**Authors:** Ran Zhang, Hai-hong Ran, Liang Peng, Fei Xu, Jun-fang Sun, Lan-ning Zhang, Yong-yan Fan, Li Peng, Geng Cui

**Affiliations:** 1 Institute of Geriatric Cardiology, Chinese People's Liberation Army General Hospital, Beijing, China; 2 Department of Geriatric Hematology, Chinese People's Liberation Army General Hospital, Beijing, China; 3 Department of Radiotherapy, Chinese People's Liberation Army 302 Hospital, Beijing, China; 4 Department of Osteology, Chinese People's Liberation Army General Hospital, Beijing, China; University of California, Merced, United States of America

## Abstract

Exposure to microgravity results in post-flight cardiovascular deconditioning and orthostatic intolerance in astronauts. Vascular oxidative stress injury and mitochondrial dysfunction have been indicated in this process. To elucidate the mechanism for this condition, we investigated whether mitochondria regulated NADPH oxidase in hindlimb unweighting (HU) rat cerebral and mesenteric arteries. Four-week HU was used to simulate microgravity in rats. Vascular superoxide generation, protein and mRNA levels of Nox2/Nox4, and the activity of NADPH oxidase were examined in the present study. Compared with control rats, the levels of superoxide increased in cerebral (*P*<0.001) but not in mesenteric vascular smooth muscle cells. The protein and mRNA levels of Nox2 and Nox4 were upregulated significantly (*P*<0.001 and *P*<0.001 for Nox2, respectively; *P*<0.001 and *P*<0.001 for Nox4, respectively) in HU rat cerebral arteries but not in mesenteric arteries. NADPH oxidases were activated significantly by HU (*P*<0.001) in cerebral arteries but not in mesenteric arteries. Chronic treatment with mitochondria-targeted antioxidant mitoTEMPO attenuated superoxide levels (*P*<0.001), decreased the protein and mRNA expression levels of Nox2/Nox4 (*P*<0.01 and *P*<0.05 for Nox2, respectively; *P*<0.001 and *P*<0.001 for Nox4, respectively) and the activity of NADPH oxidase (*P*<0.001) in HU rat cerebral arteries, but exerted no effects on HU rat mesenteric arteries. Therefore, mitochondria regulated the expression and activity of NADPH oxidases during simulated microgravity. Both mitochondria and NADPH oxidase participated in vascular redox status regulation.

## Introduction

Exposure to microgravity results in post-flight cardiovascular deconditioning and orthostatic intolerance in astronauts. The animal-based studies have confirmed the differential structural and functional changes in hindlimb unweighting (HU) rat cerebral and mesenteric arteries [1], [2]. Although much progress has been made in the past two decades, the underlying mechanism of adaptive alterations in the vasculature remains to be established.

The roles of oxidative stress injury caused by reactive oxygen species (ROS) in vascular structural and functional remodeling have been well established in cardiovascular and metabolic disorders [3], [4]. Human and rodent studies have indicated that oxidative stress occurs after spaceflight, which is more pronounced after long-duration spaceflight [5], and it might be related to oxidative/antioxidative imbalance [6]. Serum and salivary vitamin C and E levels were significantly decreased, whereas malondialdehyde levels were significantly increased in human subjects exposed to −6° head-down-tilt bed rest [7]. Dietary restrictions alleviated tissue oxidative response [8]. Microgravity affected the molecular machinery responsible for sensing alterations of flow and generated a pro-oxidative environment that activated inflammatory responses, altered endothelial behavior, and promoted senescence in cultured human umbilical vein endothelial cells [9]. Our previous work found that superoxide levels increased in HU rat cerebral arteries, which could be attributed to the activated local renin-angiotensin system [10]. We also found that NADPH oxidase inhibition with apocynin reversed abnormal vascular responses to vasoconstrictors and vasodilators, and these effects were related to the regulation of the nitric oxide synthase (NOS)-NO system in HU rat cerebral arteries [11]. However, the characteristics of expression and activity of NADPH oxidases are unknown.

NADPH oxidases have been considered the major source of ROS. The NADPH oxidase family includes seven catalytic subunits termed Nox1-5 and Duox1 and Duox2, regulatory subunits p22phox, p47phox, Noxo1, p67phox, Noxa1, p40phox, and the major binding partner Rac [12]. Of these, Nox2 and Nox4 are expressed in cardiovascular tissues and contribute to the development of cardiovascular diseases. Nox2-containing NADPH oxidase produces oxidative stress injury, which promotes vascular hypertrophy and endothelial dysfunction in cerebral arterioles [13]. Nox4 is the primary source of inflammation and tumor necrosis factor-α-induced oxidative stress leading to apoptosis in cerebral microvascular endothelial cells [14]. However, whether the expression of Nox2 and Nox4 and the activity of NADPH oxidase were altered by microgravity is unclear.

Mitochondria are the most important sources of ROS and accumulating evidence suggest of crosstalk between mitochondria and NADPH oxidase in vascular oxidative stress injury [15], [16], [17]. In addition, mitochondrial ROS (mtROS) uncouples endothelial NOS and converse xanthine dehydrogenase to the oxidase form, which constitutes crosstalk between mtROS and other sources of ROS [18]. Both Nox2 and Nox4 contribute to mtROS production [19], [20]. Nox4 and mtROS act as mediators of eNOS uncoupling triggered by Ang II in glomerular mesangial cells [20]. The inhibition of mtROS production with rotenone or myxothiazol prevented hypoxic activation of NADPH oxidase through protein kinase C (PKC)-epsilon pathway [21]. Therefore, mitochondria are not only a target for ROS produced by NADPH oxidase but also a significant source of ROS, which can stimulate NADPH oxidases [22]. In another work [23], we found that 4-week HU induced mitochondrial dysfunction and mitochondrial oxidative injury in rat cerebral vascular smooth muscle cells (VSMCs), and mitochondria-targeted antioxidant mitoTEMPO reversed mitochondrial function and attenuated mitochondrial oxidative stress injury. However, whether mitochondria regulates Nox2/Nox4 expression and the total activity of NADPH oxidases during HU are unclear.

To better understand the molecular mechanism(s) of vascular oxidative stress injury during microgravity, we detected ROS levels as well as the expression of Nox2/Nox4 and activity of NADPH oxidases in HU rat cerebral and mesenteric arteries. Mitochondria-targeted antioxidant mitoTEMPO, a mitochondria-targeted derivative of TEMPOL designed to protect mitochondria from the oxidative injury, was used to identify whether mitochondria regulated the expression and activity of NADPH oxidases during simulated microgravity.

## Materials and Methods

### Animal and Tissue Treatment

The handling and treatment of the animals were in accordance with the Guiding Principles for the Care and Use of Animals in the Field of Physiological Sciences and approved by the Chinese guidelines for experimental animals. The use and care of experimental rats were supervised and approved by Animal Ethical Committee of Chinese People's Liberation Army General Hospital.

Male Sprague Dawley rats were randomly assigned to four groups (n = 8): control (CON), HU, mitoTEMPO-treated HU (HU+MT), and mitoTEMPO-treated control (CON+MT). HU+MT and CON+MT rats received distilled water containing mitoTEMPO (0.07 mg/ml) (Alexis Biochemicals, San Diego, CA, USA) according to body weight at 0.7 mg/kg/day by gavage. The rats of the other groups received equal volume of vehicles (distilled water). The technique of HU has been previously described in detail [24] and was used to simulate microgravity in rats. The rats were housed in a 12 hour light/12 hour dark cycle and temperature (27±1°C) with *ad libitum* access to food and water.

After 28 days of treatment, each rat was anesthetized with pentobarbital sodium (40 mg/kg, intraperitoneally) and sacrificed by exsanguination via the abdominal aorta. Cerebral and mesenteric arteries were rapidly removed and placed in cold Krebs buffer solution consisting of (in mmol/L) NaCl, 118.3; KCl, 14.7; KH_2_PO_4_, 1.2; MgSO_4_·7H_2_O, 1.2; CaCl_2_·2H_2_O, 2.5; NaHCO_3_, 25; dextrose, 11.1; and EDTA, 0.026; pH 7.40.

### Reactive Oxygen Species Detection

Cellular superoxide production in cerebral and mesenteric VSMCs was assayed using dihydroethidium (DHE; Molecular Probes, Eugene, OR, USA) and high-performance liquid chromatography (HPLC) based assay to determine 2-hydroxyethidium, a superoxide-induced oxidative product of DHE. For single cell isolation, the VSMCs were dissociated from cerebral and mesenteric arteries [25], [26]. The VSMCs were washed with Krebs buffer and incubated with 10 µM DHE for 30 min at 37°C. The extra DHE was washed and Beckman HPLC System Gold model with a C-18 reverse phase column (Nucleosil 250, 4.5 mm; Sigma–Aldrich, St. Louis, MO, USA) was used to separate ethidium, dihydroethidium and 2-hydroxyethidium. Ethidium and 2-hydroxyethidium are detected with a fluorescence detector using an emission wavelength of 580 nm and an excitation of 480 nm. The 2-hydroxyethidium peak reflects the amount of superoxide formed in the VSMCs and the results were expressed as pmol/mg protein.

### Expression of NADPH Oxidase Isoforms Nox2 and Nox4

Western blot was used to identify Nox2/Nox4 protein abundance in cerebral and mesenteric arteries. The cerebral and mesenteric arteries were homogenized in 10 mM HEPES lysis buffer (containing 320 mM sucrose, 1 mM EDTA, 1 mM DTT, 10 µg/mL leupeptin, and 2 µg/mL aprotinin, pH 7.40) The lysates were then centrifuged at 12,000×*g* for 10 min at 4°C. Protein concentrations were determined with a bicinchoninic acid (BCA) assay kit (Pierce, Rockford, IL, USA). The proteins were separated by 10% SDS-polyacrylamide gels, and the fractionated proteins were electrophoretically transferred to nitrocellulose membranes (Amersham, Piscataway, NJ, USA). The membranes were incubated with mouse anti-Nox2 monoclonal (1∶1,500; Santa Cruz Biotechnology, Santa Cruz, CA, USA) or rabbit anti-Nox4 polyclonal (1∶1,000; Santa Cruz Biotechnology) primary antibodies. Membranes were incubated with goat anti-mouse or goat anti-rabbit secondary antibodies (1∶10,000; Jackson Immuno Research, West Grove, PA, USA). The enhanced chemiluminescence detection reagents (Amersham, Cleveland, OH, USA) were added, and the membranes were exposed to Hyperfilm (Amersham, Cleveland, OH, USA). An anti-GAPDH monoclonal antibody (1∶10,000; Abcam, Cambridge, MA, USA) was used to normalize for loading variations.

Real-time quantitative polymerase chain reaction (qPCR) analysis was performed using a Bio-rad IQ5 system (Bio-Rad, Hercules, USA). Total RNA was prepared by using total RNA Kit (R6934, Omega Bio-tek Inc., GA, U.S.A.) and cDNA was synthesized in cDNA Synthesis Kit (K1622, Fermentas International Inc., Canada) according to the manufacturer's instructions. Each PCR was performed in triplicate in a final volume of 20 µL solution: 10 µL of SYBR Green dye, 1 µL of diluted cDNA products, 0.2 µM of each paired primer, 8.6 µL deionized water. Protocols were as follows: initial denaturation for 10 min at 95°C, followed by 40 cycles denaturation for 15 s at 95°C and extension for 30 s at 60°C. The last cycle for dissociation of SYBR Green probe was 15 s at 95°C, 30 s at 60°C and 15 s at 95°C. The primer sequences for *Nox2* were: sense, 5′- GTGGAGTGGTGTGTGAATGCC-3′; antisense, 5′-ATGCCAGCCAACCGAGTCACA-3′; The primer sequences for *Nox4* were: sense, 5′-TAGCTGCCCACTTGGTGAACG-3′; antisense, 5′-TAGCTGCCCACTTGGTGAACG-3′; The primer sequences for *β-actin* were: sense, 5′-TGACAGGATACAGAAGGAGA-3′; antisense, 5′-TAGAGCCACCAATCCACACA-3′; Threshold cycle (Ct) values of *Nox2* and *Nox4* mRNA were measured and normalized to that of *β-actin* and expressed as a relative ratio.

### Measurement of NADPH Oxidases Activity

NADPH oxidase activity was measured using NADPH oxidase activity assay kit (Genmed Scientifics Inc., Wilmington, DE, USA) according to the manufacturer's instructions. Briefly, the cerebral and mesenteric VSMCs were washed and incubated with NADPH. NADPH oxidase activity was measured by monitoring the rate of consumption of NADPH that was inhibited by the addition of diphenyliodonium (DPI). NADPH oxidase activity was determined by spectrophotometry (Thermo Fisher Scientific Inc., Madison, WI, USA) at 340 nm and the results were expressed as % of enzyme activity compared to that of control.

### Statistical Analysis

The results are expressed as the mean±SEM. Statistical evaluation was performed by using one-way ANOVA. A *P* value <0.05 was considered statistically significant (*P<0.05, **P<0.01, ***P<0.001).

## Results

### General Data

Microgravity simulated by HU resulted in significantly lower soleus muscle mass (*P*<0.001). Soleus muscle-to-body mass ratios were significantly reduced (*P*<0.001) in HU and HU+MT rats, which confirmed the efficacy of simulated microgravity and reliability of the animal model used. Data are summarized in Table 1.

**Table 1:** Body mass (g), soleus mass (mg) and soleus:body mass ratio (mg/g) of rats from control, mitoTEMPO-treated control, hindlimb unweighting, and mitoTEMPO-treated hindlimb unweighting (n = 8 in each group).

	Initial mass	Final mass	Soleus Mass (mg)	Soleus: Body Mass, (mg/g)
CON	195.49±4.45	335.17±17.88	134.09±4.29	0.40±0.01
HU	198.69±4.76	341.35±18.25	60.59±2.49***	0.17±0.01***
CON+MT	198.18±4.38	338.12±16.96	141.11±4.12	0.40±0.01
HU+MT	199.56±4.91	341.47±18.64	79.32±3.62***	0.23±0.02***

CON, control; HU, hindlimb unweighting; CON+MT, mitoTEMPO-treated control; HU+MT, mitoTEMPO-treated HU; Values are means±SEM; ***P<0.001 vs. control.

### Effects of HU on Vascular Reactive Oxygen Species

**Figure 1:**
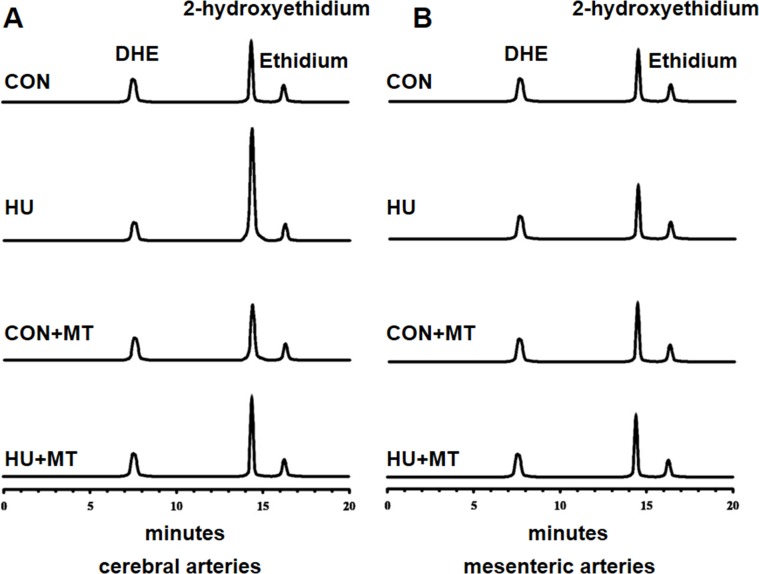
HPLC analysis of DHE-derived products in rat cerebral (A) and mesenteric (B) vascular smooth muscle cells. The cerebral and mesenteric vascular smooth muscle cells were stained with DHE. Ethidium, dihydroethidium and 2-hydroxyethidium were separated by HPLC-based method. CON, control; HU, hindlimb unweighting; CON+MT, mitoTEMPO-treated control; HU+MT, mitoTEMPO-treated HU; DHE, dihydroethidium; HPLC, high-performance liquid chromatography.

**Figure 2:**
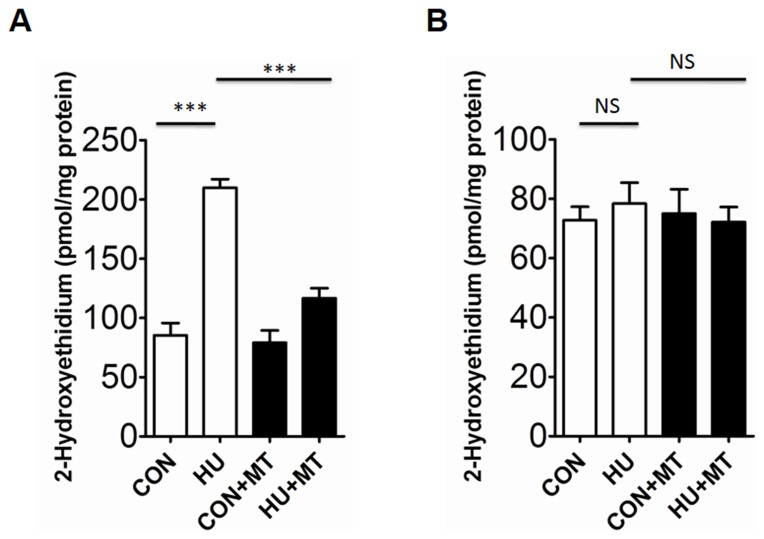
Superoxide levels in rat cerebral (A) and mesenteric (B) vascular smooth muscle cells. The superoxide generation was analyzed by HPLC-based method after staining with DHE. The 2-hydroxyethidium peak reflected the amount of superoxide formed in rat cerebral and mesenteric vascular smooth muscle cells. CON, control; HU, hindlimb unweighting; CON+MT, mitoTEMPO-treated control; HU+MT, mitoTEMPO-treated HU. DHE, dihydroethidium; HPLC, high-performance liquid chromatography. Values are mean±SEM. ***P<0.001 for HU *vs*. control or HU+MT.

To investigate whether HU altered the redox status of rat cerebral and mesenteric arterial VSMCs, we detected superoxide levels using DHE and HPLC-based method (Figure 1 and Figure 2). Four-week HU rat cerebral VSMCs showed significantly higher levels of superoxide than their age-matched controls (*P*<0.001), and chronic treatment with the mitochondria-targeted antioxidant mitoTEMPO significantly attenuated superoxide generation (*P*<0.001) (Figure 1A and Figure 2A). However, HU or mitoTEMPO did not show any statistically significant effect on superoxide generation in mesenteric arteries (Figure 1B and Figure 2B).

### Effects of HU on Expression of Nox2 and Nox4

**Figure 3:**
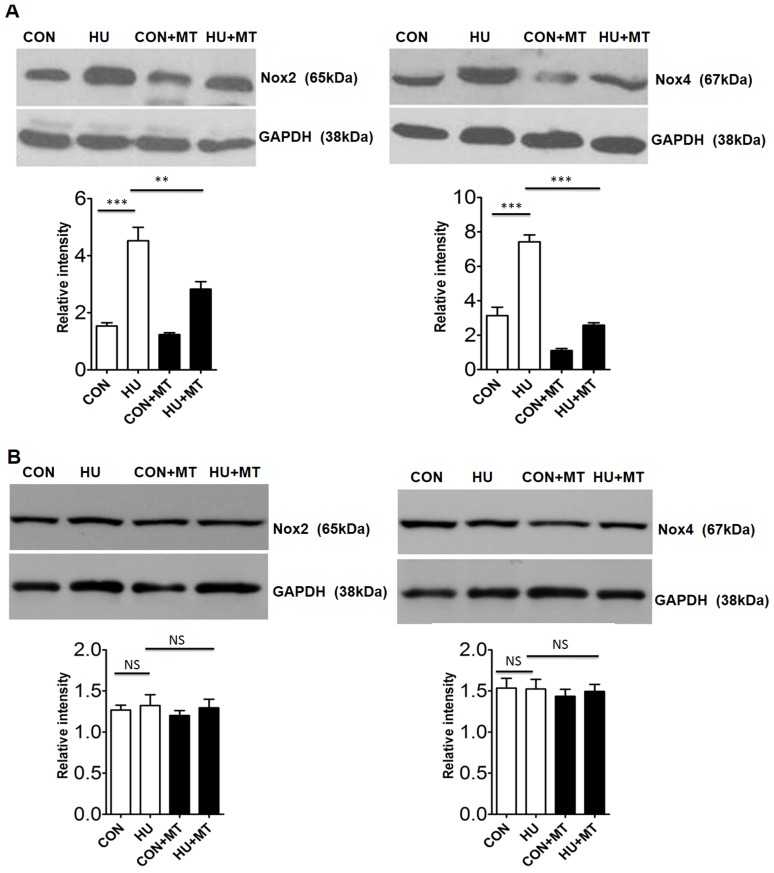
The protein expression of Nox2 and Nox4 in rat cerebral (A) and mesenteric (B) arteries. The protein abundance of Nox2 and Nox4 was analyzed by western blot. CON, control; HU, hindlimb unweighting; CON+MT, mitoTEMPO-treated control; HU+MT, mitoTEMPO-treated HU. Values are mean±SEM. **P<0.01 for HU *vs.* control, ***P<0.001 for HU *vs*. HU+MT.

**Figure 4:**
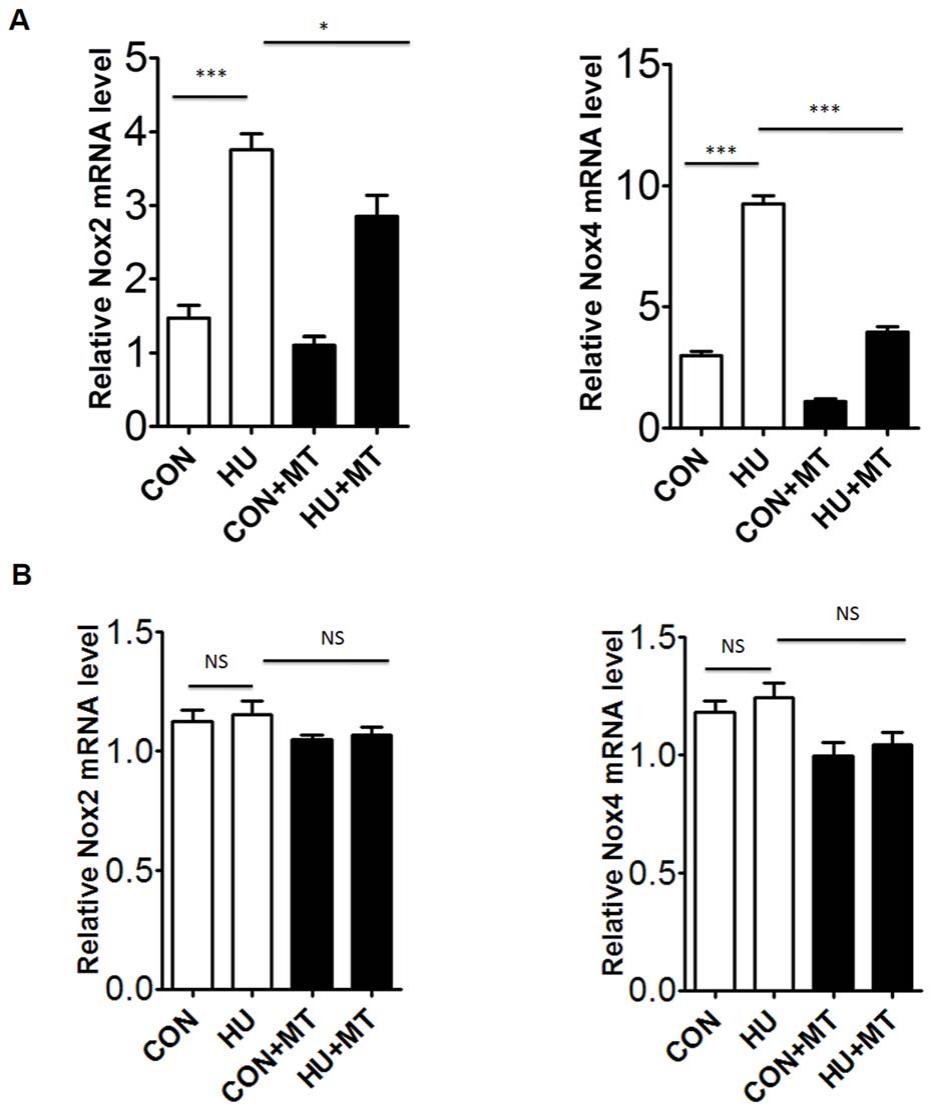
The mRNA expression of Nox2 and Nox4 in rat cerebral (A) and mesenteric (B) arteries. The mRNA abundance was analyzed by quantitative polymerase chain reaction. CON, control; HU, hindlimb unweighting; CON+MT, mitoTEMPO-treated control; HU+MT, mitoTEMPO-treated HU. Values are mean±SEM. *P<0.05 for HU vs. HU+MT and ***P<0.001 for HU *vs*. control for *Nox2*, ***P<0.001 for HU *vs.* control or HU+MT for *Nox4*.

We detected the expression of Nox2 and Nox4 in HU rat cerebral and mesenteric arteries by western blot and qPCR (Figure 3 and Figure 4). The protein and mRNA levels of Nox2 increased significantly (*P*<0.001 and *P*<0.001, respectively) in HU rat cerebral arteries compared with the control (Figure 3A; Figure 4A), whereas Nox2 expression did not vary in mesenteric arteries between control and HU rats (Figure 3B; Figure 4B). The protein and mRNA levels of Nox4 were also significantly higher in HU rat cerebral arteries (*P*<0.001 and *P*<0.001, respectively) (Figure 3A; Figure 4A), whereas their expression levels did not vary in mesenteric arteries (Figure 3B; Figure 4B). As expected, mitoTEMPO restored both the protein and mRNA expression levels of Nox2/Nox4 in cerebral arteries (*P*<0.01 and *P*<0.05 for Nox2, respectively; *P*<0.001 and *P*<0.001 for Nox4, respectively), whereas it had no effects on Nox2/Nox4 in mesenteric arteries.

### Effects of HU on NADPH Oxidases Activity

**Figure 5:**
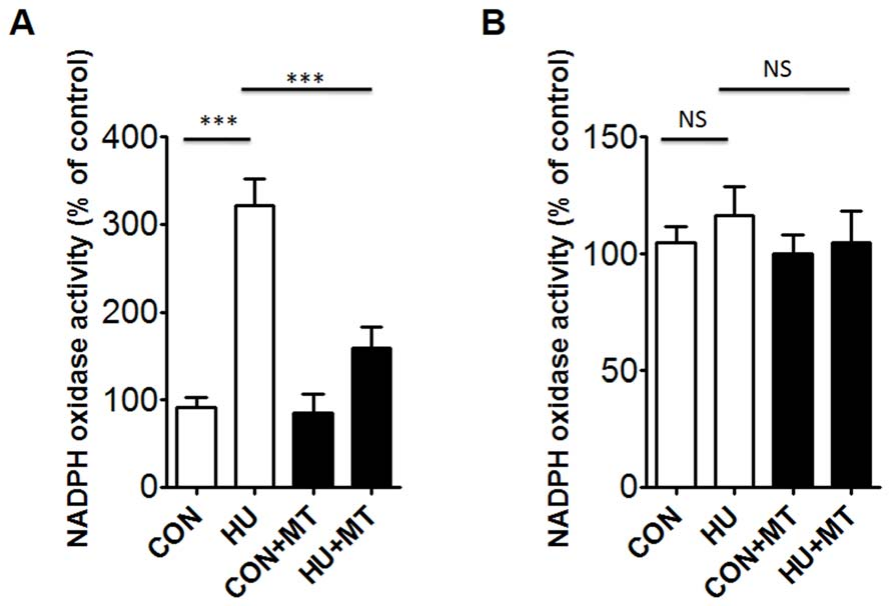
NADPH oxidase activity in rat cerebral (A) and mesenteric (B) vascular smooth muscle cells. CON, control; HU, hindlimb unweighting; CON+MT, mitoTEMPO-treated control; HU+MT, mitoTEMPO-treated HU. Values are mean±SEM. ***P<0.001 for HU *vs*. control or HU+MT.

To determine whether NADPH oxidase activation was involved in cerebrovascular oxidative stress injury, we measured NADPH oxidase activity (Figure 5). Treatment of rats with 4-week HU activated NADPH oxidase in cerebral arterial VSMCs (*P*<0.001). By contrast, HU had no stimulatory effect on NADPH oxidase activity in mesenteric VSMCs. We also determined the effects of mitoTEMPO on vascular NADPH oxidase activity and found that mitoTEMPO decreased NADPH oxidase activity in HU rat cerebral arteries (*P*<0.001), whereas mitoTEMPO did not affect NADPH oxidase activity in HU rat mesenteric arteries.

## Discussion

The present study provided, for the first time to the best of the authors' knowledge, three important findings: (1) mitochondria-targeted antioxidant mitoTEMPO attenuated HU rat cerebrovascular oxidative stress injury; (2) mRNA and protein expression levels of Nox2 and Nox4 increased in HU rat cerebral arteries, which were restored by mitoTEMPO; and (3) the total activity of NADPH oxidases increased in HU rat cerebral arteries, which was restored by mitoTEMPO.

Cardiovascular deconditioning complicates manned spaceflight and leads to post-flight orthostatic intolerance and decreased working capacity in astronauts [1], [2]. In spaceflight and ground-based human studies, the investigators have gained insights that gravitational pressure gradients on earth do not exist in the circulation and cerebral blood flow is maintained by a higher blood pressure [27], and these fluid and pressure gradient shifts result in differential vascular structural and functional adaptive changes [1], [2], [27]. The evidence from rodent models showed that contractile responses to vasoconstrictors are enhanced, and endothelium-dependent relaxation is attenuated in HU rat cerebral arteries. However, in HU rat mesenteric arteries, vascular responses to both vasoconstrictors and vasodilators are attenuated [1], [2]. As for the underlying mechanism(s), vascular inflammation, oxidative stress injury, adaptive changes in ion-channels, and the NOS-NO system have been indicated in the vascular functional remodeling during HU [1], [2], [10], [11], [28]. We postulated that vascular oxidative stress injury was an underlying mechanism of changes in the NOS-NO system because of the increased superoxide levels in HU rat cerebral arteries [10]. As expected, NADPH oxidase accounts for the enhanced vascular superoxide production and impaired endothelium-dependent relaxation of HU rat cerebral arteries, and NADPH oxidase inhibition with apocynin reverses the vascular responses to vasoconstrictors and vasodilators [11]. In this work, we confirmed the increased cytoplasmic superoxide production in 4-week HU rat cerebral VSMCs using DHE probe, which was attenuated by mitoTEMPO. Although oxidative stress injury in HU rat cerebral arteries is confirmed during HU, the molecular mechanism(s) remains to be established.

Superoxide production by NADPH oxidases is implicated in the pathogenesis of cardiovascular diseases [29]. The NADPH oxidases are critical mediators of cardiovascular physiology and pathophysiology, which includes seven catalytic subunits termed Nox1-5 and Duox1 and Duox2, regulatory subunits p22phox, p47phox, Noxo1, p67phox, Noxa1, p40phox, and the major binding partner Rac [12]. The NADPH oxidase isoforms Nox1, 2, 4, and 5 are expressed in the vasculature and are different in the activity, responses to stimuli, and type of ROS released [30]. Nox2 and Nox4 regulate the redox status in cerebral arteries [13], [14]. We have provided the first evidence that not only the expression of Nox2/Nox4 but also the total activity of NADPH oxidases were increased in 4-week HU rat cerebral arteries. These results could better explain why NADPH oxidase inhibition with apocynin restored impaired endothelium-dependent relaxation in our previous work [11]. Nox2 promotes the development of endothelial dysfunction, hypertension, and inflammation, and Nox4 protects the vasculature during stress [31], [32]. The Nox4 might function as an inducible Nox isoform because of close correlation between Nox4 mRNA and ROS generation [33]. The overexpression of endothelial Nox4 exerts vasodilation, which is attributable to increased H_2_O_2_ production and decreased NO inactivation [34]. These data seem contrary to Lee's that Nox4 contributed to ROS generation in both cytoplasm and mitochondria triggered by Ang II [20]. Superoxide derived from the Nox2 isoform plays important roles in angiotensin II-mediated inward remodeling [35] and promotes hypertrophy and causes endothelial dysfunction in cerebral arterioles, possibly involving interaction with NO [13]. Nox2 mutation exhibits a significant increase in forearm-mediated vasodilation with increased NO bioavailability [36], [37]. Nox2 deficiency protects against hypercholesterolemia-induced impairment of neovascularization, which is linked to decreased ROS production [38]. A recent study by Wenzel and co-contributors [39] found that Nox2 in infiltrating monocytes and macrophages was also accounts for angiotensin II-induced vascular dysfunction and arterial hypertension. Taken together, these data suggest of complicated roles of NADPH oxidases in the pathogenesis of cardiovascular diseases. We have demonstrated that NADPH oxidase inhibition with apocynin restored the expression and the activity of NADPH oxidases in another work (data did not show here); however, the roles and regulatory mechanism of Nox2/Nox4 (including the other isoforms) remain to be established.

The molecular regulation of NADPH oxidases has been well documented [40], [41]; however, the mechanisms by which NADPH oxidase is regulated in the cardiovascular system are still unclear. The accumulating evidence suggested that there are interactions between NADPH oxidases and mitochondria in vascular oxidative stress [15], [16], [17], and the stimulation of mitochondria increases the activity of NADPH oxidases, which enhances cellular ROS production [22]. Redox communications between mitochondria and NADPH oxidases constitute an important form of ROS-induced ROS release and is involved in a variety of physiological signaling pathways in the vasculature [42]. As for the underlying mechanisms of the crosstalk between mitochondria and NADPH oxidase, opening of mitoKATP channels in rat VSMCs depolarized the mitochondrial membrane potential and increased cellular superoxide levels [43]. MtROS-induced NADPH oxidase activation could be also prevented by inhibitors of the mPTP, PKC, tyrosine kinase cSrc, NADPH oxidases and be amplified by mitochondrial superoxide dismutase deficiency, which led to endothelial dysfunction in angiotensin II-induced hypertension [44]. Except for mitochondrial superoxide dismutase, glutathione peroxidase-1 (GPx-1) may also be involved in vascular oxidative stress and cerebrovascular functional remodeling in HU rat because aged GPx-1(-/-) mice displayed more prominent decreased NO bioavailability and endothelial dysfunction through eNOS uncoupling [45]. Mitochondria-targeted antioxidants decreased mitochondrial superoxide, inhibited total cellular superoxide, reduced cellular NADPH oxidase activity, and restored the level of bioavailable NO [46]. We found that apocynin and mitoTEMPO reversed mitochondrial dysfunction induced by HU and decreased mitochondrial ROS levels in HU rat cerebral VSMCs in another work [23]. To investigate whether mitochondria regulate NADPH oxidases during HU, we used mitoTEMPO as the mitochondria-targeted antioxidant and found that mitoTEMPO restored protein and mRNA expression levels of Nox2/Nox4 isoforms, and the activity of NADPH oxidases. Taken together, these data are very important because they suggest mitochondrial regulation of NADPH oxidases and interplay between mitochondria and cytoplasmic NADPH oxidases in cerebrovascular oxidative stress during simulated microgravity.

As for the HU rat mesenteric arteries, neither the expression of Nox2/Nox4 nor the total activity of NADPH oxidases was altered. Although different changes in VSMCs ion channels [19], [20] secondary to gravitational pressure gradient shifts in the circulation [21] have been indicated in differential vascular structural and functional remodeling during microgravity, the underlying mechanism remain to be established. Our present data did not support the roles of NADPH oxidases in functional changes of mesenteric arteries. Further studies are needed to elucidate this issue.

Based on our findings, simulated microgravity upregulated the expression of NADPH oxidases isoforms Nox2/Nox4 and increased the total activity of NADPH oxidases, which were restored by mitochondria-targeted antioxidant mitoTEMPO. Crosstalk between cytoplasmic NADPH oxidases and mitochondria was observed in HU-induced cerebrovascular oxidative stress injury. Further studies are needed to investigate the expression of NADPH oxidase isoforms other than Nox2/Nox4 and identify their individual contributions to NADPH oxidase activity and roles in vascular functional remodeling during microgravity.

## Supporting Information

Checklist S1 **ARRIVE Checklist.**
(DOC)
